# Awareness Regarding Pap Smear Among Female University Students of Karachi: A Cross-sectional Survey

**DOI:** 10.7759/cureus.2784

**Published:** 2018-06-11

**Authors:** Meeshal Khan, Amara Zafar, Ramsha Muneer, Amna A Siddiqui

**Affiliations:** 1 Dow Medical College, Civil Hospital Karachi, Dow University of Health Sciences, Karachi, PAK; 2 Dow Medical College, Civil Hospital Karachi, Dow University of Health Sciences, Karachi, Pakistan

**Keywords:** pap smear, cervical cancer, women, reproductive age, awareness

## Abstract

Objective: To determine the knowledge and practices regarding Papanicolaou (Pap) smear test among female university students of Karachi.

Method: This cross-sectional study was carried out among 491 female university students of Karachi. Participants were selected by random sampling between the ages of 18 to 30 years; from different universities namely Dow University of Health Sciences (DUHS), Jinnah Sindh Medical University (JSMU), and Institute of Business administration (IBA). Pearson chi-square test and Mann-Whitney U tests were applied as the primary statistical methods.

Results: Out of 491 participants, three-quarters of the participants knew about the Pap smear test. However, only 1.6% had undergone the procedure. Age, socioeconomic status (SES), and medical background all had a significant relationship with the awareness of Pap smear test. One-third of the females in the study blamed lack of knowledge as the major reason for not getting a Pap smear while another third blamed the lack of recommendation by health care professionals.

Conclusion: According to our study, female undergraduate students of Karachi have insufficient knowledge and exposure to Pap smear, a screening test that could decrease the burden of cervical cancer among the female population of our country. Further research is required to assess the severity of the problem and consequently strategize to control it.

## Introduction

Cervical cancer was previously ranked the second most common female cancer worldwide [1] but has now declined to the fourth most common female cancer [2]. However, the burden of the disease is now limited primarily to the less developed regions of the world, where one-third of the cervical cancer burden is attributed to South Asia [3]. Most of the data in Pakistan regarding cervical cancer is based on regional cancer registries, or studies conducted at private institutions [4-5]. Therefore, there is a paucity of data regarding cervical cancer incidence in Pakistan, which makes it difficult to assess the true burden of the disease in the region, making it a seemingly impossible task to plan awareness, screening, and prevention strategies to combat this growing problem.

The significant decline in the incidence of cervical cancer in the developed world can be credited largely to the use of the Papanicolaou (Pap) smear as a screening tool for cervical cancer caused by human papilloma virus (HPV) [6]. HPV is transmitted sexually and persistent genital infection with HPV is the primary risk factor for cervical cancer [7]. The Pap smear is an efficient tool to detect precancerous and cancerous changes in the cervix caused by HPV at an early stage, and despite its limitations, the effect of Pap smear testing on reducing the mortality of cervical cancer is a breakthrough in gynecological health [6]. 

Cervical cancer is a significant cause of mortality in this region primarily because of late presentation [4]. Consequently, it is safe to say that the awareness about cervical cancer and its risk factors in the general population is negligible, and the practice of getting a Pap smear done regularly is even lower, particularly in the population belonging to the lower socio-economic background with limited formal education [8]. There is a deficiency of knowledge regarding Pap smear testing amongst health care workers as well, which is to be expected in a country like Pakistan where there is a poor health care set up [9].

As cervical cancer is likely to be caused by sexually transmitted HPV, it is important to consider the cultural and religious beliefs especially in Muslim communities, where they could have a possible negative impact on practices and attitudes regarding Pap smear testing [4].

The success of Pap smear testing in the developed world is largely based on extensive screening programs and the awareness regarding the test’s importance in preventing cervical cancer [10]. A low socio-economic country like Pakistan, which is already burdened with problems of infectious diseases and malnourishment, is deficient in healthcare infrastructure and budget that is required to carry out nation-wide awareness and Pap smear screening programs to reduce the burden of this disease.

With these parameters in consideration, this cross-sectional study aims to assess the knowledge, attitude, and practices regarding Pap smear among university going females of Karachi. The study in hand is a positive step towards evaluating the magnitude of the problem and the efforts needed to tackle it.

## Materials and methods

This cross-sectional study was conducted on the knowledge, awareness, and attitude of female university students in Karachi towards Pap smear. It was carried out from 1st May 2017 to 22nd July 2017 after approval from the Institutional Review Board of Dow University of Health Sciences (DUHS).

Participants were selected by random sampling between the ages of 18 to 30 years; from different universities namely DUHS, Jinnah Sindh Medical University (JSMU), and Institute of Business administration (IBA). Even though the Pap smear test is recommended at the age of 21, we could not ignore the phenomenon of young marriages in Pakistan, which leads to many young women being married off at the tender ages of 18-21. They come into contact with the organism earlier than most, hence, the need for them to be aware of the Pap smear test. A total of 491 university going females were enrolled in the study.

A structured questionnaire was designed after thorough literature searches. The questionnaire was thoroughly reviewed by two proficient doctors and pilot tested on 10 students for relevance, coherence, and clarity before being administered to the study. The questionnaire was applied by interpersonal interviews conducted by fourth-year medical students. The sample size was calculated in OpenEPI software sample size calculator with 95% confidence interval and 5% margin of error.

The questionnaire comprised of two parts. The first part included questions on demographic data, for example, age, marital status, education, and income. Participants with a monthly income of 50,000 PKR were categorized as high socioeconomic status (SES) and participants with income less than 50,000 PKR were categorized as low SES. Women were also inquired of their marital status and sexual activity. The second part consisted of knowledge about Pap smear and their practices regarding the HPV vaccines and Pap smear. Participants were questioned regarding their concerns for not choosing to get Pap smear done.

Data were analyzed descriptively using IBM Statistical Package for the Social Sciences (IBM SPSS Statistics for Windows, Version 20.0 Armonk, New York) and tables were constructed using Microsoft Excel 2016. Chi-square test was applied to test the significance of differences between categorical variables, and Mann-Whitney U was applied to check the significant differences between categorical and continuous variables of non-parametric data. Results for the qualitative variable were presented as frequencies and percentages whereas results for continuous data were presented as mean and standard deviation. P value was considered significant if less than 0.05.

## Results

Table 1 shows the mean age of our participants as 21.58 (SD 1.98). 94.9% of them were single while 5.1% were married. In terms of SES, 13.2% had a monthly income of <50,000 PKR and were thus classified as low SES while 86.8% belonged to a high SES (monthly income >50,000 PKR). Majority of the women (82.1%) belonged to the medical field in undergraduate universities. Three quarters of them claimed to know about the Pap smear procedure (74.5%). Almost all the participants (98.8%), however, denied having undergone the procedure and only 1.6% had previously opted for a Pap smear. 91.2% of them agreed that they would consult a doctor if they experienced symptoms related to their genitals.

**Table 1:** Demographics and sample characteristics 50,000 PKR = 432 USD Pap: Papanicolaou

Characteristics	N (%)
Age	Mean 21.58 std. deviation 1.98 Median 21.0 Inter-quartile range 20.0-22.0
Marital status	
Single	466 (94.9)
Married	25 (5.1)
Socio-economic Status	
Family income <50,000 PKR/month (Low)	65 (13.2)
Family income >50,000 PKR/month (High)	426 (86.8)
University Field	
Medical	403 (82.1)
Non-medical	88 (17.9)
Sexually active	
Yes	41 (8.4)
No	450 (91.6)
Do you know about Pap smear	
Yes	366 (74.5)
No	125 (25.5)
Have you undergone Pap smear	
Yes	6 (1.2)
No	485 (98.8)
Would you consider consulting a doctor if you had genital symptoms?	
Yes	448 (91.2)
No	43 (8.8)

Table 2 shows only 8.4% of all women reported to be sexually active while the rest (91.6%) denied any sexual activity. The relationship between age and knowledge of Pap smear was found to be significant (p value=<0.001). A significant association with knowledge of Pap smear was found with SES (p value=0.004) where 89.4% women belonging to high SES had knowledge of Pap smear while only 10.6% of the women belonging to low SES claimed to know about the procedure. Similarly, association between a medical background of education and knowledge of Pap smear was also significant (p value=<0.001) where 91.3% individuals belonging to medical background had knowledge while only 8.7% of people from non-medical backgrounds claimed to have knowledge of Pap smear. On the contrary, vaccination status against HPV and sexual activity seemed to have no link with knowledge about Pap smear (p values 0.345 and 0.217, respectively).

**Table 2:** Factors associated with knowledge about Pap smear Pap: Papanicolaou

	Do you know about Pap smear?		
Characteristics	Yes	No	p-value
Age			<0.001
Socioeconomic status			0.004
Less than 50,000	39/366 (10.6)	26/125 (20.8)	
More than 50,000	327/366 (89.4)	99/125 (79.2)	
Field of Education			<0.001
Medical	334/366 (91.3)	69/125 (55.2)	
Non- medical	32/366 (8.7)	56/125 (44.8)	
Vaccination Status			.345
Yes	21/366(5.7)	9/125 (7.2)	
No	345/366 (94.3)	116/125 (92.8)	
Sexually Active			.217
Yes	28/366 (7.7)	13/125 (10.4)	
No	338/366 (92.3)	112/125 (89.6)	

**Figure 1:**
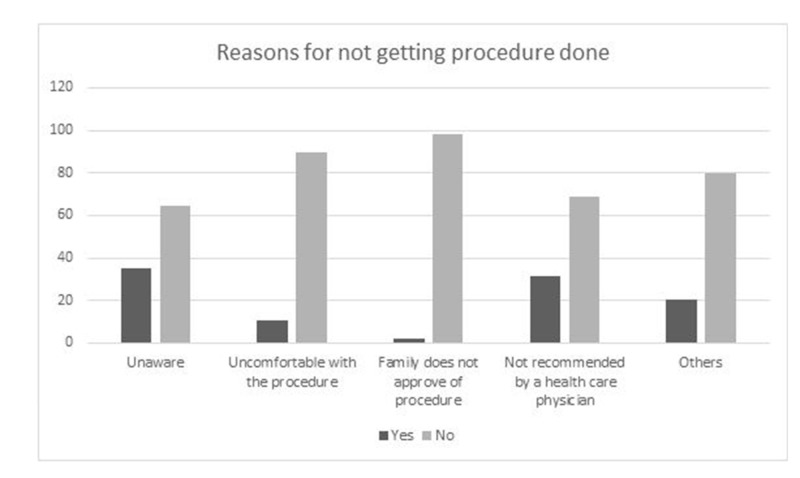
Reasons for not getting Pap smear test Pap: Papanicolaou

Figure 1 shows that the most common reason among all women for not getting a Pap smear done was unawareness about the procedure (35.2%), followed by a lack of recommendations by heath care providers (31.2%). 10.5% of participants reported that they feel uncomfortable about the procedure and 1.8% cited family’s disapproval as the reason for not opting for Pap smear. The remaining participants (20.4%) chose ‘other reasons’ and did not choose any of the aforementioned options.

## Discussion

In terms of screening modalities for HPV, Pap smear is an effective as well as efficient method to detect cervical carcinomas and dysplastic changes in the cervix uteri, especially in the early stages [6]. In our study, 344 out of 491 participants (75.1%) had heard of the Pap smear test while only five women (0.01%) had undergone this test. Furthermore, 91.2% of females reported that they were likely to consult a doctor for a checkup or screening regarding cervical cancer. The degree of awareness and practice of Pap test in our study is lower than expected, as most of the participants included in the study were from medical universities. Similarly, Ismail H et al. reported that only 9% women were attending gynecology clinics for their annual gynecological examination and only 13.7% of women had Pap smear done in their life [11]. Findings of a study from India were no different where although 58.6% knew about the availability of screening tests, only 3% had opted for a Pap smear [12]. These are alarmingly low rates of screening statistics among women of a region in which cervical cancer prevalence appears to be clearly dominant. However, these statistics are to be expected in this region since there is a severe lack of education, particularly amongst females, superimposed on socio-cultural and religious beliefs and practices which create further barriers for women to access proper health-care. A similar example of social-cultural hinderance to the regular practice of Pap smear test is seen in the women of United Arab Emirates, despite having a positive attitude towards it [13].

A study conducted in Canada explored the relationship of formal education and level of acculturation with awareness and practices regarding cervical cancer and its screening by Pap test, among South Asian and Tamil women residing in Canada [14]. The study found low levels of knowledge and practice of cervical cancer and Pap smear testing in the South Asian and Tamil women and were significantly related to lower levels of formal education and lack of acculturation in these women. This research reaffirms our findings regarding the role of education and awareness as major contributory factors to prevalence of Pap smear testing.

Furthermore, the study in hand found lesser amount of knowledge and utilization of Pap smear in women from lower socio-economic backgrounds as compared to their counterparts. These findings are compatible with those from a study conducted in Botswana [15]. This is an expected result since access to health care is depleted in low socioeconomic countries like Pakistan. Likewise, almost all the women in an Indian study showed willingness to get vaccination against HPV, if it was provided free of cost [12]. This goes on to show how important a role easy access to health-care can play to help prevent deadly disease like cervical cancer.

Another interesting finding from our study included the relationship of age and knowledge of Pap smear test. Similarly, Monica Ideström’s study of Denmark showed that knowledge and concerns regarding Pap smear test were dependent on age, where younger women showed lesser degree of awareness and practice as compared to the older participants [16].

Secondary to the lack of awareness regarding Pap smear testing, lack of recommendation by health care providers was a common cause contributing to the paucity of the practice of Pap smear testing. A study conducted in three major hospitals of Karachi depicted an alarming situation where only 37% of the interns and nursing staff recognized Pap smear as a screening test for cervical cancer. This problem stems from the grass root level, beginning from medical institutions were emphasis on public health awareness is significantly lacking [9].

One of the major limitations of our study was the selection of sample size used, which was small, selective, and unevenly distributed. Replication of this study with a greater sample size and more participants form non-medical universities can even provide a comparison between medical and non-medical students regarding Pap smear awareness.

## Conclusions

According to our study, female undergraduate students of Karachi have insufficient knowledge and consequent exposure to Pap smear, a screening test that could decrease the burden of cervical cancer among the female population of our country. Our study suggests that comprehensive and focused awareness campaigns regarding pap smear and it's role in cervical cancer screening, along with a regular recommendation by physicians, can greatly enhance the practice of obtaining routine pap smear tests. Further research is required to assess the severity of the problem and consequently strategize to minimize it, preferentially on a national level with widespread awareness campaigns and screening programs.
